# Rapid-onset respiratory failure caused by diabetic ketoacidosis complicated with pulmonary mucormycosis: a case report and literature review

**DOI:** 10.3389/fmed.2026.1850830

**Published:** 2026-05-18

**Authors:** Li He, LiLi Chen, Cheng Liu

**Affiliations:** Department of Critical Care Medicine, Dazhou Central Hospital, Dazhou, Sichuan, China

**Keywords:** case report, diabetic ketoacidosis, pulmonary mucormycosis, rhizopus, therapeutic strategy

## Abstract

**Objective:**

To investigate the rapid progression and key treatment strategies for invasive pulmonary mucormycosis caused by Rhizopus in patients with diabetic ketoacidosis (DKA).

**Methods:**

We report a fatal case of severe Rhizopus pneumonia in a patient with DKA and conducted a systematic review of relevant cases from PubMed and Web of Science for descriptive analysis.

**Results:**

A 39-year-old male with DKA developed severe lung lesions within days post-admission. Despite confirmation via pathological diagnosis and the initiation of liposomal amphotericin B therapy, the patient succumbed rapidly. Incorporating 15 cases identified from the literature, the total cohort comprised 16 patients, yielding an overall mortality rate of 25% (4/16). Cases involving DKA frequently exhibited narrow diagnostic windows and rapid progression to respiratory failure. Most cases (11/15) received amphotericin B-based therapy, and some with localized lesions (7/15) underwent surgical resection.

**Conclusion:**

Pulmonary mucormycosis with DKA can progress rapidly and carries high risk. Treatment typically involves amphotericin B preparations, supplemented by surgery for focal disease. Key strategies include maintaining a high index of clinical suspicion in high-risk hosts, early pathological/molecular diagnosis, prompt antifungal therapy alongside metabolic correction, and timely surgical evaluation for localized lesions.

## Introduction

Pulmonary mucormycosis is a rare but highly lethal invasive fungal infection caused by fungi of the order Mucorales, and its incidence has increased over the past few decades (1). The disease occurs in individuals with severely impaired immune function, such as patients with hematological malignancies, solid organ transplant recipients, and individuals with diabetes (2). Among them, diabetic ketoacidosis (DKA) is considered one of the most important risk factors, and its mechanism is potentially attributed to hyperglycemia, acidosis-induced inhibition of phagocytic function, and increased free iron levels (3, 4).

Fungi of the order Mucorales (e.g., Rhizopus) are the most common pathogen of pulmonary mucormycosis, characterized by an acute onset and rapid progression. However, its clinical manifestations lack specificity, often leading to misdiagnosis and delayed treatment; consequently, mortality rates can exceed 76% (5–7). Once it progresses to disseminated infection, the mortality rate is 96%. Currently, the diagnosis relies on the discovery of characteristic hyphae by histopathology and confirmation by culture or molecular biological methods (8). In recent years, metagenomic next-generation sequencing (mNGS) and other technologies have offered new possibilities for early diagnosis (9). For treatment, international guidelines recommend liposomal amphotericin B as the cornerstone of therapy and emphasize the importance of early surgical intervention (8, 10). However, for patients with rapidly progressive disease who cannot tolerate surgery, the treatment strategy still faces great challenges.

However, clinical experience in diagnosing and treating this disease remains generally limited. This article reports a case of rapidly progressive pulmonary Rhizopus infection in a patient with DKA, and aims to explore the heterogeneity in the clinical course of such infection under different immune backgrounds through a comprehensive literature review, in order to provide reference for early identification and intervention of critical cases.

## Case report

The patient was a 39-year-old male miner. He was transferred to our hospital’s Intensive Care Unit on November 21, 2024, presenting with “chest tightness and shortness of breath for 3 days, with worsening and increased dyspnea for 6 h.”

### History of present illness and treatment timeline

On November 17, 2024, the patient experienced chest tightness and shortness of breath of unknown etiology, accompanied by a cough productive of a small volume of yellowish-white sticky phlegm. The patient was diagnosed with “common cold” at a local clinic and received symptomatic treatment (paracetamol and a Chinese patent medicine for cold relief, but systemic glucocorticoids were not used), but the symptoms did not improve.

On November 20, 2024, the patient was treated at the endocrinology department of the local county people’s hospital. The venous blood glucose was 44 mmol/L and the blood ketone was > 8 mmol/L. The diagnosis was “DKA and pneumonia.” Subsequently, meropenem was administered for antimicrobial therapy (1 g, q8h, intravenous guttae), insulin was used to lower blood sugar, and fluid resuscitation was initiated.

On November 21, 2024, the patient experienced a worsening of symptoms with shortness of breath and a decrease in oxygen saturation to 80%, accompanied by altered consciousness. Immediate oxygen therapy via face mask (10 L/min) was administered, and a bedside arterial blood gas test revealed severe acidosis and hypoxemia (pH 7.27, PaO2 45 mmHg, Lac 4.12 mg/dL). The patient’s D-dimer was significantly elevated at 1,844 ng/mL. The high-sensitivity cardiac troponin T (cTnThs) level was mildly elevated at 0.024 μg/L, and the pro-B-type natriuretic peptide (proBNP) level was markedly elevated at 4,531 pg/mL. Due to a sudden and severe deterioration of the patient’s condition, with the consent of the family, emergency tracheal intubation and invasive mechanical ventilation (FiO_2_ 100%) were performed at the local hospital. The patient presented with septic shock, requiring norepinephrine at a maximum dose of 1.8 μg/kg/min and metaraminol at 2.8 μg/kg/min to maintain a mean arterial pressure above 65 mmHg. After discussion among the family members, the patient was transferred to our department.

### Past medical history

The patient has a 4-year history of type 2 diabetes, characterized by irregular treatment and suboptimal glycemic control. He has a 6-year occupational history in underground mining. He has a smoking history spanning over 20 years, with a consumption of approximately 10 cigarettes per day. The patient’s family reports no history of chronic respiratory diseases, including chronic obstructive pulmonary disease (COPD), asthma, or pneumoconiosis.

### On admission physical examination

Vital signs: Temperature 36.8°C, heart rate 124 beats per minute, respiratory rate 16 breaths per minute (ventilator-controlled), blood pressure 122/68 mmHg (norepinephrine 2.1 μg/kg/min and metaraminol 3.2 μg/kg/min). The Sequential Organ Failure Assessment (SOFA) was 12, and the Acute Physiology And Chronic Health Evaluation II (APACHE II) score was 26, indicating severe illness. The patient was deeply sedated (RASS score, -4), orally intubated, and receiving mechanical ventilation. The ventilator was set to synchronized intermittent mandatory ventilation (SIMV) mode with a rate of 16 breaths per minute, a fraction of inspired oxygen (FiO_2_) of 100%, a positive end-expiratory pressure (PEEP) of 10 cmH_2_O, and a pressure support (PS) of 10 cmH_2_O. On pulmonary examination, percussion revealed dullness over both lung fields, and bilateral moist rales were audible, more pronounced on the right side. The remainder of the physical examination was unremarkable.

### Auxiliary examination

#### Laboratory tests

Arterial blood gas analysis on admission (FiO2 100%) showed pH 7.099, PaO2 54 mmHg, and oxygenation index 54 mmHg. White blood cell count was 7.58 × 10^9^/L, with a neutrophil percentage of 84.9%, and C-reactive protein was 330.4 mg/L.

#### Imaging examination

**FIGURE 1:**
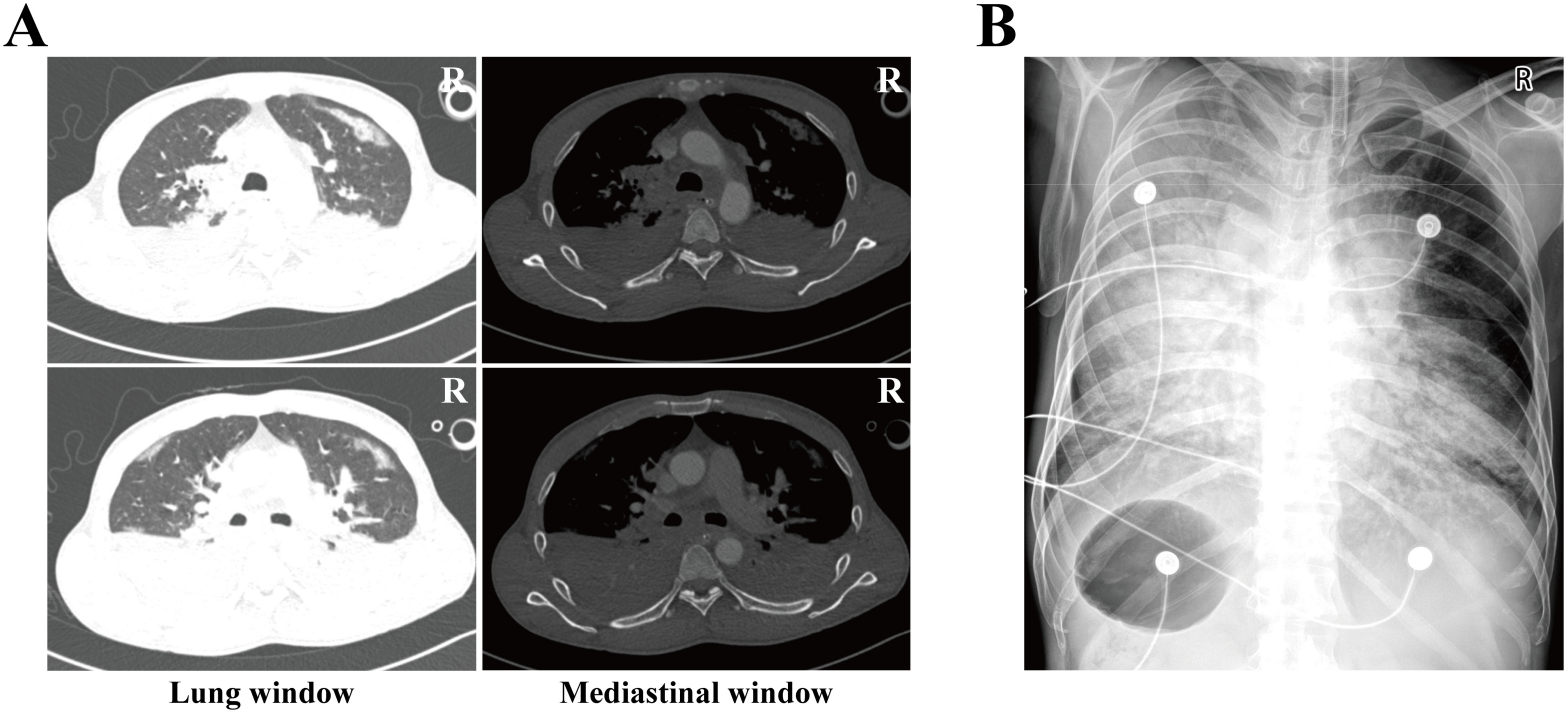
Imageological examination. **(A)** Chest CT on admission. **(B)** Bedside DR two days after admission. CT Computed Tomography, DR digital radiography.

Chest computed tomography (CT) on admission showed multiple patchy consolidation shadows in both lungs (Figure 1A). In order to observe the distribution and characteristics of the lesions in a more three-dimensional and continuous manner, the continuous axial dynamic images of this CT examination (lung window and mediastinal window) have been provided as Supplementary Videos 1, 2. A follow-up bedside digital radiograph (DR) 2 days later demonstrated rapid progression and fusion of the bilateral lung opacities (Figure 1B).

#### Endoscopy

**FIGURE 2:**
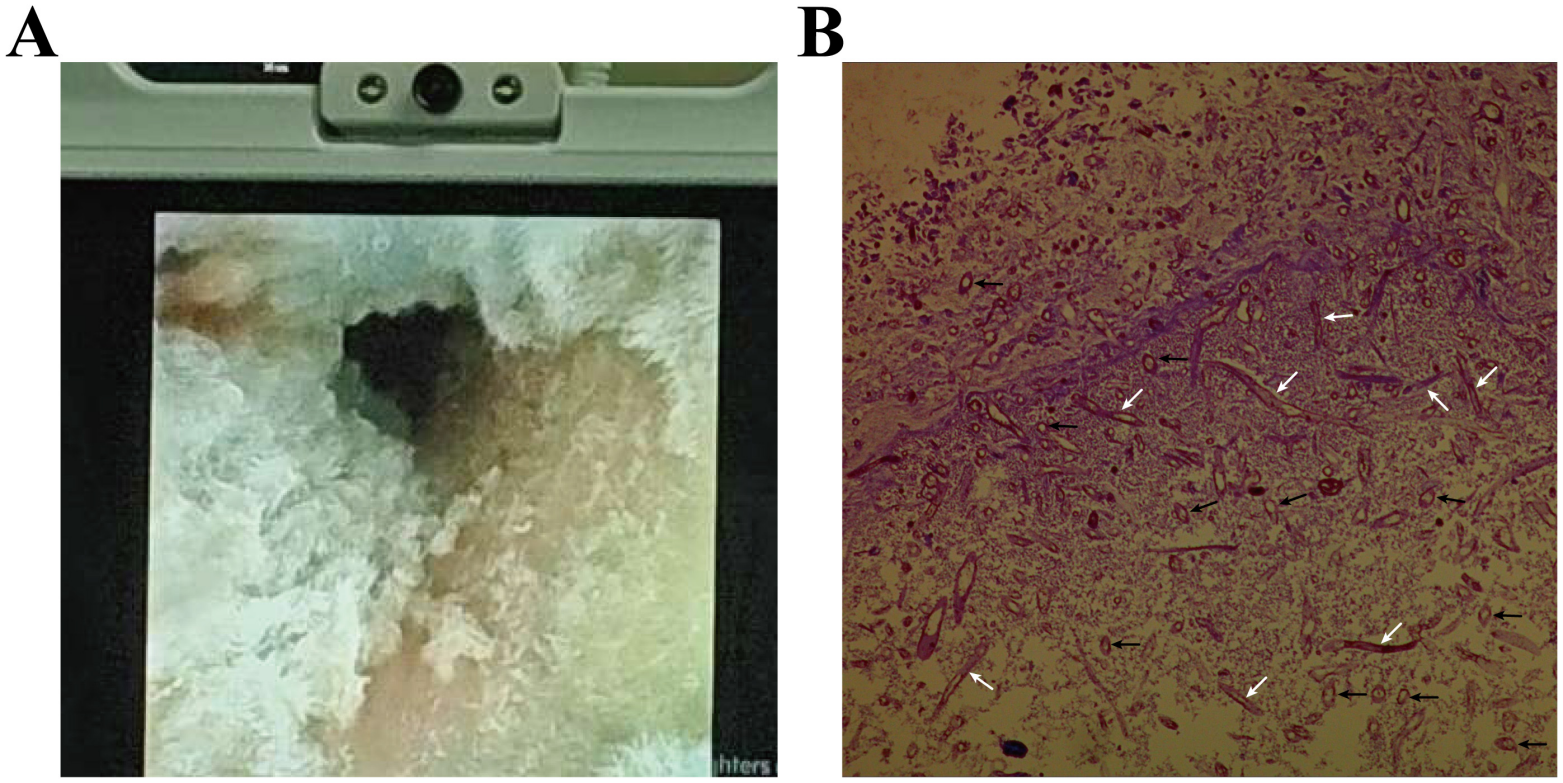
Bronchoscopy and pathological biopsy. **(A)** Typical bronchoscopic view. **(B)** Biopsy histopathology (H&E stain, × 200), White arrow: mycelium; Black arrow: spore.

Bronchoscopy showed that the airways were extensively covered with white necrotic debris and villous material (Figure 2A). In addition, video of bronchoscopy has been provided as Supplementary Video 3, showing the morphology of lesions in the airways dynamically.

#### Pathogen tests

Sputum specimens sent for examination ruled out common viral infections such as avian influenza and influenza. Aerobic and anaerobic blood cultures were negative. Respiratory secretions collected with a protected specimen brush were cultured on two consecutive occasions, and filamentous fungi were identified. Histopathological examination of biopsy tissues revealed abundant fungal spores and hyphae (Figure 2B). The final mNGS results confirmed that the causative pathogen was Rhizopus (Supplementary Table 1).

### Diagnosis and treatment

#### Initial diagnosis

Severe pulmonary infection, respiratory failure and shock.

#### Initial treatment

Upon admission, the patient was immediately given invasive mechanical ventilation, deep sedation, and prone position ventilation. Anti-shock treatment included active fluid resuscitation (approximately 3,000 mL of crystalloid fluid), combined use of metaraminol and norepinephrine to maintain blood pressure, empirical antimicrobial therapy [meropenem (1 g, q8h, intravenous guttae) combined with linezolid (600 mg, q12h, intravenous guttae) and doxycycline (100 mg, q12h, intravenous guttae)], and hydrocortisone succinate (100 mg, q12h, intravenous guttae) for anti-inflammation. Concurrently, albumin and gamma globulin were administered.

#### Antifungal treatment

Based on the bronchoscopic biopsy pathology findings highly suggestive of mucormycosis, antifungal treatment was immediately initiated (Liposomal amphotericin B, 100 mg (approximately 3 mg/kg), qd, intravenous guttae. Treatment start date: November 22, 2024). The patient had no history of prophylactic or therapeutic antifungal drug use prior to this episode.

#### Final diagnosis

Confirmed invasive pulmonary mucormycosis caused by Rhizopus (Diagnosis date: November 23, 2024, based on mNGS results).

### Outcome

Despite the initiation of antifungal treatment, the patient’s respiratory and circulatory failure progressed irreversibly. After being informed of the critical condition and Extracorporeal Membrane Oxygenation (ECMO) therapy as an option, the patient’s family—considering the extremely poor prognosis and limited expected therapeutic benefit—opted to forgo further treatment. The family voluntarily discharged the patient.

### Disease course timeline chart

**FIGURE 3:**
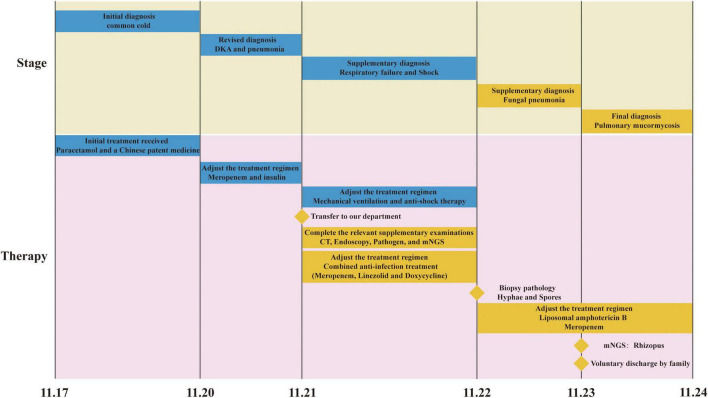
Timeline of the clinical course. Blue: outpatient/external diagnosis & treatment; yellow: in-hospital diagnosis and treatment. Key events from the initial common cold (11/17) to final diagnosis of pulmonary mucormycosis and voluntary discharge (11/24) are shown. CT Computed Tomography; DKA Diabetic Ketoacidosis; mNGS metagenomic Next-Generation Sequencing.

To illustrate the disease progression and interventions, a timeline of the clinical course was developed (Figure 3).

## Discussion

To explore the clinical characteristics of such infections, we performed a narrative literature review as described below. In the PubMed database, we employed a combination of Medical Subject Headings (MeSH) and free-text terms using the following search string: (“Rhizopus”[MeSH] OR “Rhizopus”[Title/Abstract]) AND (“Mucormycosis”[MeSH] OR “mucormycosis”[Title/Abstract]) AND (“Lung Diseases, Fungal”[MeSH] OR “pulmonary”[Title/Abstract] OR “lung”[Title/Abstract]). For the Web of Science database, we conducted a topic search using an adapted strategy: (“Rhizopus” OR “Rhizopus species”) AND (“Mucormycosis” OR “zygomycosis”) AND (“Lung Diseases, Fungal” OR “Pneumonia” OR “pulmonary” OR “lung”). The inclusion criteria were: (1) case reports or series; (2) confirmed pulmonary mucormycosis caused by Rhizopus; (3) studies providing complete clinical information. The exclusion criteria were: (1) non-human studies; (2) non-pulmonary infections; (3) pathogens other than Rhizopus; (4) unavailability of the full text or presence of only an abstract without full data. Finally, 15 literature cases that met the analysis criteria were included for descriptive summary (Table 1) (11–23). The average age of the patients was 47.8 years, and 73.3% were male. Diabetes mellitus and renal transplantation related immunosuppression were the most common host background (Table 2).

**TABLE 1:** Basic information of patients with pulmonary mucormycosis caused by Rhizopus.

Author/publication time	Age/gender	Underlying diseases	Occupational exposure	Time from onset to diagnosis	Diagnosis method	Lobectomy/antifungal agents	Outcome
Latif et al. (11)	57/M	Kidney transplant, DM	NA	NA	Bronchoscopy (KOH + culture)	Yes/Amphotericin B	Survived
Latif et al. (11)	43/M	Kidney transplant, DM	NA	NA	Bronchoscopy (KOH + culture)	Yes/Amphotericin B	Survived
Demirag et al. (12)	52/M	Kidney transplant, DM	NA	∼3 weeks	BAL culture	Yes/Amphotericin B	Survived
Lee et al. (13)	52/M	Kidney transplant, DM	NA	4 weeks	BAL histology + culture	Yes/Liposomal Amphotericin B	Survived
Chacko et al. (14)	45/M	Kidney transplant	NA	∼3 months	Open lung biopsy (histology)	No/Liposomal Amphotericin B, Voriconazole	Survived
Li et al. (15)	47/F	Kidney transplant, DM	NA	1 month	Abscess fluid culture	Yes/Liposomal Amphotericin B	Survived
Kwan et al. (16)	61/M	Kidney transplant, Post-transplant DM	NA	∼2 months	Lobectomy + tissue culture	Yes/Posaconazole	Survived
Navarro Vergara et al. (17)	56/F	Kidney transplant, DM	NA	NA	Fine needle aspiration + PCR	No/Amphotericin B	Died
Compain et al. (18)	42/F	DM	NA	∼1 month	Lung biopsy + culture + molecular	Yes/Liposomal Amphotericin B, Posaconazole	Died
Ukoha et al. (19)	57/M	Kidney transplant, DM	NA	4 weeks	Bronchial biopsy + culture	No/Amphotericin B	Died
Yuan et al. (20)	26/M	DM	Carpenter	1 month	Tissue biopsy + culture + molecular	No/Posaconazole	Survived
Cheng et al. (21)	34/M	DM	NA	2 days	Bronchoscopy biopsy + mNGS	No/Amphotericin B cholesterol sulfate complex	Improved
Wang et al. (22)	37/F	DM	NA	1 month	EBUS-TBNA + histology + mNGS	No/Amphotericin B, Posaconazole, Nebulized and EBUS-guided Amphotericin B	Improved
Wang et al. (23)	61/M	Kidney transplant, DM, COVID-19	NA	19 days	BALF mNGS	No/Oral Isavuconazole	Survived
Wang et al. (23)	47/M	Kidney transplant, COVID-19	NA	∼13 days (after COVID-19 neg)	BALF mNGS, sputum culture	No/Oral Isavuconazole	Survived

DM, Diabetes Mellitus; BAL, Bronchoalveolar Lavage; mNGS, Metagenomic Next-Generation Sequencing; EBUS-TBNA, Endobronchial Ultrasound-guided Transbronchial Needle Aspiration.

**TABLE 2:** Clinical characteristics of 15 patients with pulmonary mucormycosis caused by Rhizopus.

Feature	Number (%) of all patients (*n* = 15)	Mortality, n/N (%)
Mean age, y	47.8 (range 26–61)	
Gender		
Male	11/15 (73.3%)	1/11(9.1%)
Female	4/15 (26.7%)	2/4 (50.0%)
Underlying diseases		
Diabetes mellitus	4/15 (26.7%)	1/4(25.0%)
Solid organ transplant (kidney)	2/15 (13.3%)	0/2 (0%)
Both	9/15 (60.0%)	2/9 (22.2%)
Diagnosis method		
Histopathology and/or culture	13/15 (86.7%)	2/13 (15.4%)
Molecular (mNGS/PCR)	5/15 (33.3%)*	2/7 (28.6%)
Therapy		
Antifungal agents		
Amphotericin B formulation	11/15 (73.3%)	3/11 (27.3%)
Azole monotherapy (posaconazole/isavuconazole)	3/15 (20.0%)	0/4 (0%)
Amphotericin B followed by Azole	3/15 (20.0%)	1/3(33.3%)
Lobectomy		
Lobectomy and antifungal agents	7/15 (46.7%)	1/7 (14.3%)
Antifungal agents only	8/15 (53.3%)	2/8 (25.0%)
Outcome		
Survived/improved	12/15 (80.0%)	
Died	3/15 (20.0%)	

mNGS, metagenomic next-generation sequencing; PCR, polymerase chain reaction.

Based on the literature review and the present case, although the clinical manifestations of pulmonary mucormycosis lack specificity, the progression of the disease shows significant heterogeneity under different immune backgrounds. The data from this body of literature showed that the infection in the context of DKA often progressed to respiratory failure rapidly. However, in chronically immunocompromised hosts such as transplant recipients, the diagnostic latency may be longer and localized lesions are more common on imaging (11, 14, 19). This difference may be due to acidosis and hyperglycemia in the DKA environment, which creates a microenvironment conducive to invasion for mucormycosis (24). In contrast, although the transplant recipient is immunosuppressed, regular medical surveillance may allow the disease to be detected at a relatively localized stage. Therefore, for patients with DKA, the window period for clinical identification and intervention is extremely short, and clinicians must maintain a high index of suspicion.

In this series, 13 cases (86.7%) were confirmed by traditional histopathology or culture, which is still the cornerstone of diagnosis. However, in rapidly progressive cases, waiting for results from conventional approaches may delay treatment decisions. Notably, 7 cases (46.7%) in this group utilized molecular diagnostic techniques such as mNGS or Polymerase Chain Reaction (PCR), which all provided early indications of the pathogen, thus prompting timely adjustments to treatment strategies (9). The data suggest that establishing a parallel diagnostic mode of “morphology + molecular biology” may be key to overcoming the diagnostic bottlenecks associated with DKA complicated by severe pneumonia (25). Drawing insights from this case series, the traditional “treatment after diagnosis” strategy may lead to the loss of the therapeutic window for “very high-risk” patients with DKA and pulmonary infiltrates. While differences exist in the application scenarios and levels of evidence for molecular methods (mNGS/PCR), inferring their overall diagnostic performance solely from this case series is challenging. Nevertheless, for high-risk hosts exhibiting rapid radiographic progression and unresponsiveness to conventional antimicrobial therapy, a more pragmatic strategy involves obtaining samples as early as possible to establish a parallel evidence chain of “pathology/culture plus molecular detection.” This approach facilitates the timely adjustment of treatment plans upon identification of the pathogen.

Regarding treatment strategies, the current international guidelines recommend combination therapy (8). Of the 15 cases reported in the literature in this group, 11 were treated with amphotericin B preparations. Of these, seven patients underwent lobectomy combined with antifungal therapy, resulting in a mortality rate of 1/7 (14%) in this subgroup. Eight patients received medical therapy alone, and the mortality rate in this group was 2/8. This distribution suggests that surgical clearance may be associated with better outcomes in patients with relatively limited lesions who are amenable to surgery, and is consistent with the reported advantages of surgical intervention in reducing the fungal burden (26). However, this finding must be viewed with caution, as it is likely influenced by case selection bias and should not be used for causal inference. Therefore, the more important clinical implication is to advance the timing of surgical evaluation; specifically, ensuring that the feasibility of surgery is discussed by the multidisciplinary team when the infection is relatively limited, rather than reserving surgery as a salvage intervention for end-stage disease.

A distinguishing characteristic of this case is the rapid progression to respiratory failure in the context of severe DKA, which further confirms the important role of the metabolic environment in the aggressive progression of Rhizopus pulmonary infection. In addition to the suppression of immune cells by hyperglycemia and ketone bodies, elevated free iron levels resulting from acidosis may also promote fungal growth (24). The severe metabolic disturbance present in this patient on admission provided an ideal substrate for fungal invasion. This suggests that rapid correction of acidosis and hyperglycemia is equally critical to antifungal therapy in the treatment strategy. In addition, although there is no direct pathological evidence to confirm a diagnosis of overt silicosis based on the patient’s occupational history (long-term underground mining), the possibility of impaired alveolar macrophage function caused by silica dust cannot be completely excluded (27). This potential factor warrants consideration in assessing the susceptibility of similarly high-risk patients.

Our study has certain limitations. Most of the included literature consisted of case reports, which were subject to publication bias and may overestimate the success rate of treatment. The small sample size (*n* = 15) limited the performance of multivariate regression analysis, and the effect of confounding factors on prognosis could not be eliminated. Some cases lacked specific genotype information of strains.

In conclusion, patients with DKA and concurrent Rhizopus pulmonary infection may present with a narrower window for recognition and intervention. The core management strategy for high-risk hosts includes high vigilance, concurrent diagnosis, rapid correction of metabolic disorders, early initiation of amphotericin B liposome-based therapy, and advanced multidisciplinary surgical evaluation of localized lesions. Since this study is based on a single case report and a small sample of literature, the above findings still need to be further verified by larger studies.

## Data Availability

The original contributions presented in this study are included in this article/Supplementary material, further inquiries can be directed to the corresponding author.

## Supplementary material

Supplementary Video S1 CT lung window.

Supplementary Video S2 CT mediastinal window.

Supplementary Video S3 Fiberoptic bronchoscopy.
