# Supplementary material for: Rapid-onset respiratory failure caused by diabetic ketoacidosis complicated with pulmonary mucormycosis: a case report and literature review
Source: Front Med (Lausanne). 2026 May 18;13:1850830. doi: 10.3389/fmed.2026.1850830 (PMC13222958; doi:10.3389/fmed.2026.1850830)
Supplement: Supplementary file 1 [file Table_1.docx]

Supplementary Table 1. Report of Pathogen mNGS. mNGS Metagenomic Next-Generation Sequencing.

| **Genus** | | | | **Complex/Species** | | |
| --- | --- | --- | --- | --- | --- | --- |
| **Type** | **Name** | **Sequence**  **Number** | **Relative**  **Abundance** | **Name** | **Sequence**  **Number** | **Relative**  **Abundance** |
| Fungus | Rhizopus | 672272 | 99.03% | Rhizopus oryzae | 622160 | 99% |
